# Twin pregnancy in a bicornuate uterus in rural Kenya: A case report for accidental discovery and successful delivery

**DOI:** 10.4102/phcfm.v12i1.2198

**Published:** 2020-07-21

**Authors:** Hussein E. Elias, James A. Amisi

**Affiliations:** 1 Department of Family Medicine, College of Health Science, Moi, University, Eldoret, Kenya

**Keywords:** twin pregnancy, bicornuate uterus, rural Kenya, low-middle income country, Mullerian malformations, APGAR score

## Abstract

**Introduction:**

Uterine anomalies are often identified during pregnancy, during infertility evaluation or pregnancy miscarriage and have been associated with an increased risk of adverse pregnancy outcomes. Although some studies have documented the rare occurrence of spontaneous twin pregnancy in each horn of a bicornuate uterus, this is the first time this is being documented in Kenya, to the best of our knowledge. This is a rare occurrence and reporting of this case adds to the documentation of such cases.

**Patient presentation:**

This is a case report for a 30-year-old female, para 2+0 at 34 weeks 4 days by dates, who presented with features of labour. Upon examination, she had normal vital signs and a fundal height of 38 weeks with multiple foetal parts both in cephalic presentation and two foetal heart rates within normal range. Her antenatal profile was non-contributory and had undergone two ultrasounds that confirmed twin gestation with no other notable findings.

**Management and outcome:**

The patient had a spontaneous vertex delivery of the first twin with a good outcome. There was a delay in the delivery of the second twin and a caesarean section was done with an indication of non-reassuring foetal status and low-lying placenta. The bicornuate uterus was accidentally identified during the surgery. The outcome was good, with an APGAR score of 6 in the first minute and 9 at 10 min.

**Conclusion:**

Although this is a rare occurrence, we would like to sensitise healthcare workers in rural low- to middle-income countries that this can occur, and they should attempt to increase antenatal diagnosis as it can influence the mode of delivery.

## Introduction

Mullerian duct anomalies are congenital uterine disorders resulting from abnormal formation and fusion of the Mullerian ducts during organogenesis.^1^ The Mullerian ducts form the fallopian tube, uterus, cervix and upper two-thirds of the vagina, which together comprise the female reproductive tract.^2^ Failure of the Mullerian duct fusion during organogenesis results in a bicornuate or didelphys uterus.^2^ Congenital uterine anomalies have an estimated global occurrence of 0.1% to 3%, and 15% to 25% of women have been associated with an increased rate of adverse foetal outcomes, miscarriages, preterm delivery, premature rupture of membranes, foetal malpresentation and problems with fertility.^1,2,3^ Uterine anomalies often are identified during antenatal visits, during infertility evaluation or pregnancy miscarriage.^4^ Case reports have shown an extremely rare occurrence of spontaneous twin pregnancy in a bicornuate uterus.^3,4^

We report an accidental discovery and a successful delivery case of a rare occurrence of spontaneous twin pregnancy in uterus bicornis with each twin in each horn of the uterus.

## Patient presentation

A 30-year-old gravid woman arrived at the labour ward in a level 4 hospital in rural Western Kenya with complains of intermittent lower abdominal pains that had begun 4 h before arrival in the hospital. The pains were increasing in intensity and frequency and were associated with blood-stained, non-foul-smelling mucoid vaginal discharge. She was received by the midwife at the maternity ward and a brief history and physical examination was done.

She was a gravida 3 para 2+0. Her last normal menstrual period had been on 20 April 2018 and a month later she had self-diagnosed the pregnancy by using a test kit at home. Her first antenatal visit had been on 22 August 2018 (gestational age 18 weeks) and she had attended a total of four antenatal clinic (ANC) visits. During her first visit, her ante-natal profile was: haemoglobin of 10.9 g/dL, blood group A, rhesus positive, Human Immunodeficiency Virus (HIV) negative and a syphilis test: Venereal Disease Research Laboratory (VDRL)-non-reactive. She reported having had an uneventful pregnancy. Medications received through the pregnancy were iron and folate, which she had taken throughout the pregnancy. She had received tetanus toxoid injections, malaria prophylaxis and mebendazole. During the antenatal period two ultrasound imaging scans had been done at the same facility. Her first ultrasound had been on 24 September 2018 (gestational age 22 weeks) and the report had been as follows: sonographic features suggestive of live twin intrauterine pregnancy, both in cephalic presentation, with gestational age estimated at 21 weeks 6 days as per the bigger baby. The second ultrasound had been done a month later on 24 October 2018 and the report had been as follows: sonographic features suggestive of live twin intrauterine pregnancy, both in breech presentation, with gestational age of 27 weeks 4 days as per the bigger baby. Her obstetric and gynaecological history was non-contributory. She reported having been experiencing normal menstrual periods with no history of pregnancy miscarriages. Her first pregnancy and delivery had been in 2010 and had been uneventful. She had attended her ante-natal clinics but no ultrasound imaging had been done then. She had delivered at home a live male infant weighing 3.2 kg. Her second pregnancy and delivery had been in 2014 and had been uneventful at a health facility. She had attended the ante-natal clinic but no imaging had been done. She had delivered a live female infant weighing 3.3 kg.

Upon her arrival at the facility, the midwife examined her. Her vital signs were as follows: blood pressure 123/69 mm/Hg and a pulse of 78 beats per minute (bpm). Abdominal exam revealed a fundal height of 38 weeks with multiple foetal parts felt and two foetal heads felt in cephalic presentation. Two foetal heart sounds were heard of 136 bpm and 138 bpm respectively, which were regular. A vaginal examination was also performed and revealed a cervical dilation of 5 cm, soft in consistency at mid-position and more than 50% effaced with bulging membranes.

The patient was admitted with an assessment of twin pregnancy, both in cephalic presentation at 34 weeks 4 days by dates and 38 weeks by fundal height in the latent phase of labour.

## Management and outcomes

One and a half hours after admission and five and a half hours later, after onset of lower abdominal pain, she delivered a live male infant weighing 2.3 kg with an APGAR score of 10 in the first minute, 10 at 5 min and 10 at 10 min (APGAR is an acronym made up of the initial letters of Appearance, Pulse, Grimace, Activity, and Respiration – vital signs tested for immediately after birth). Two and a half hours later she had not delivered the second twin and the doctor on call was informed. The doctor evaluated the patient and made an assessment of retained second twin with a possible low-lying placenta (felt during the vaginal examination) and non-reassuring foetal status. A decision for caesarean delivery of the second twin was made.

**FIGURE 1:**
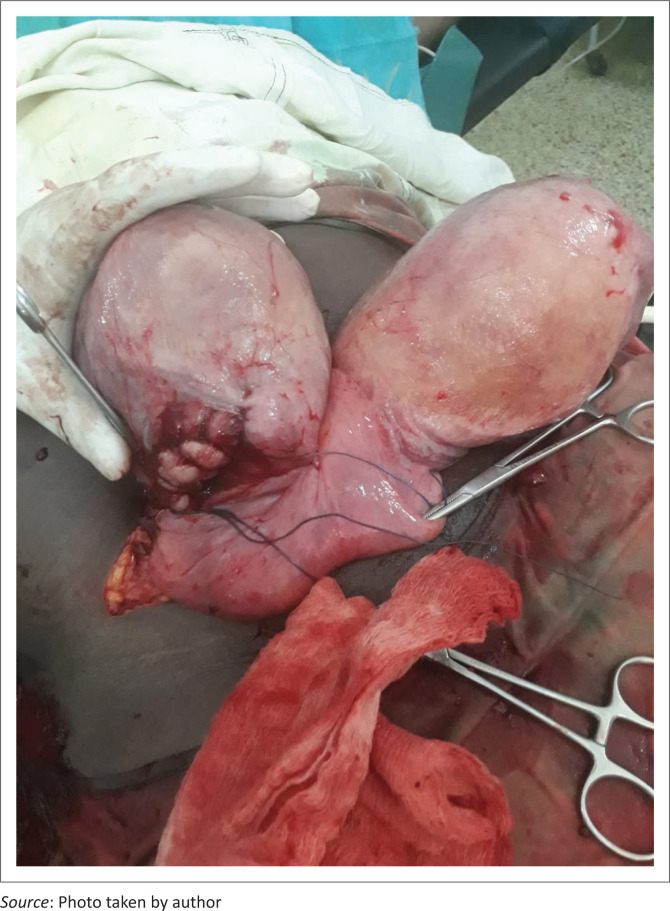
After insertion of final haemostatic sutures.

During the surgery, after opening the abdomen in layers, a gravid uterus was identified located on the right side of the abdomen. A lower uterine incision was made and the observations were as follows: low-lying placenta with a foetus in cephalic presentation. The foetus was delivered, followed by the placenta. Immediately after delivering the placenta, the other horn of the bicornuate uterus spontaneously popped out of the abdomen. Closure of the incision site was done and haemostasis was achieved. The abdomen was closed in layers. She received 4 L of intravenous normal saline fluid intraoperatively and one unit of whole blood by transfusion immediately after surgery owing to estimated blood loss of 2. The blood loss included the loss during the vaginal delivery of the first twin and the subsequent caesarean section. The blood loss of 2 L was considered post-partum haemorrhage (PPH) and managed as per the hospital’s guidelines for PPH management. Four hours later, in the post-operative period, the patient was fully awake and well, with normal vital signs (see Figure 1).

The second twin was a live male infant weighing 1.7 kg with an APGAR score of 6 in the first minute, 7 at 5 min and 9 at 10 min. The baby was transferred to the newborn unit for observation. The infant was well and started breastfeeding within 24 h after delivery. The mother had an uneventful post-operative period.

On the fifth post-operative day, the mother was discharged with her twins, who were in good health condition.

### Ethical consideration

Verbal and written consent to publish this was obtained from the patient and an exemption letter from the Institutional Research and Ethics Committee (IREC) of Moi University with reference number 0003355 was issued.

## Discussion

Uterine malformations are not uncommon; they occur as a consequence of abnormal organogenesis, fusion or canalisation of Mullerian ducts during foetal development.^1^ Although the prevalence is unknown in Kenya, these malformations occur in about 5.5% to 6.6% of the general population globally.^1,5^ The prevalence is much higher in women with infertility (7.3%) and recurrent miscarriage (16.7%).^5^ Examples of such malformations of the genital tract include double uterus with or without a double cervix and vagina, bicornuate uterus or separate uterus and/or abnormalities of the genital tract, cervix, vagina and urinary tract.^6^

Patients with uterine malformations and multiple gestation experience a significant risk of complications over and above patients without malformations in both the mother and the unborn babies.^7^ These complications include: foetal malpresentation, low birth weight, rupture of the uterus and labour abnormalities, including, but not limited to, dynamic dystocia.^7,8,9^ Although there is significant risk of complications, *some women have uneventful pregnancies and have been reported to deliver successfully without complications*.^10^ This can explain the history of previous uncomplicated pregnancies and vaginal deliveries in this case report.

Bicornuate uterus has a global prevalence of about 0.4% and results from failure of Mullerian duct fusion at the apex of the uterus, leading to two uterine horns.^1,6^ The diagnosis of a bicornuate uterus confers a higher risk of miscarriage, preterm delivery and malpresentation than a didelphys uterus.^11,12^

There are a few documented studies of women with uterine malformations and twin pregnancies.^7^ A search on PubMed revealed that the earliest documentation was by Duraisamy in 1926^13^ and Bhagwat SA in 1953.^14^ In this case, although the antenatal ultrasound was done and there is a history of previous uncomplicated deliveries, the diagnosis of bicornuate uterus was not made. In this context, the possibility of few documented cases could be because of having uncomplicated deliveries and the possibility of missing the diagnosis. Furthermore, there may be low interest in reporting rare cases. The low reporting makes it unnecessary to have standard guidelines on managing such cases with resultant poor pregnancy outcomes, given the high incidences of poor outcomes associated with uterine malformations.

Adams et al. in a more recent case report in 2019 recommended ongoing publication of case reports with respect to uterine malformations and twin pregnancy to provide a larger body of data for what is a rare presentation.^15^ Similarly, Ahluwalia et al. had noted in 2014 that there are no specific guidelines for management and so recommended maintenance of a worldwide register to report cases and consistently improve pregnancy outcomes and foetal survival rates.^16^ It is in response to these concerted calls that we report the above case. To the best of our knowledge, this is the first case that has been reported in Kenya, a Lower Middle-Income Country (LMIC). The case also highlights the possibility of missing a diagnosis of bicornuate uteri prenatally in LMICs where availability and access to quality obstetric ultrasound and other diagnostic modalities are not always guaranteed. In order not to miss this rare occurrence, there should be further training for ultra-sonographers working in rural settings so that they can recognise it. It should be emphasised to healthcare workers working in the antenatal clinic that they should improve on their prenatal diagnosis capabilities of uterine malformations. Since there are no specific guidelines for management, there should be concerted efforts to develop specific guidelines for management by various stakeholders to enable other healthcare workers who will encounter such cases to manage with ease, with favourable maternal and foetal outcomes.
